# A Decade of HIV Pre-Exposure Prophylaxis (PrEP) Cascade Monitoring: National Trends and Persistent Gaps in PrEP Use Among Gay, Bisexual and Queer Men and Non-Binary People in Australia

**DOI:** 10.1007/s10461-026-05100-x

**Published:** 2026-03-17

**Authors:** James MacGibbon, Daniel Storer, Timothy R. Broady, Sarah K. Calabrese, Anthony K. J. Smith, Curtis Chan, Johann Kolstee, Simin Yu, Jeanne Ellard, Dean A. Murphy, James Gray, Limin Mao, Lucy Watson, Jane Costello, John de Wit, Benjamin R. Bavinton, Martin Holt

**Affiliations:** 1 Centre for Social Research in Health, UNSW Sydney, Sydney, NSW Australia; 2 Department of Psychological and Brain Sciences, George Washington University, Washington, DC USA; 3 School of Population Health, Curtin University, Perth, WA Australia; 4 Collaboration for Evidence, Research and Impact in Public Health, Curtin University, Perth, WA Australia; 5 Kirby Institute, UNSW Sydney, Sydney, NSW Australia; 6 Australian Research Centre in Sex, Health & Society, La Trobe University, Melbourne, VIC Australia; 7 School of Population Health, UNSW Sydney, Sydney, NSW Australia; 8 New South Wales Ministry of Health, Sydney, NSW Australia; 9 Health Equity Matters, Sydney, NSW Australia; 10 ACON, Sydney, NSW Australia; 11 Positive Life NSW, Sydney, NSW Australia; 12 Department of Interdisciplinary Social Science, Utrecht University, Utrecht, Netherlands

**Keywords:** HIV PrEP, Prevention cascade, Care continuum, Men who have sex with men, Sexual minority men, Disparities

## Abstract

We have monitored Australia’s maturing HIV pre-exposure prophylaxis (PrEP) program for the past decade, providing a unique view of long-term implementation among gay, bisexual and queer men and non-binary people. Using two national surveys, the GBQ + Community Periodic Surveys (GCPS, 2014–2023, N = 78,378) and the PrEPARE Project (2017–2023, N = 5,278), we constructed and iteratively refined cascades to monitor PrEP suitability, awareness, use, and service engagement. The PrEPARE cascade also included willingness, provider engagement and psychosexual benefits of PrEP. By 2023, both cascades showed near-universal PrEP awareness among PrEP-suitable participants. In the GCPS cascade, PrEP use increased from < 1% of PrEP-suitable participants in 2014 to 30% in 2023, while in PrEPARE it increased from 21% in 2017 to 46% in 2023. However, in 2023, 29% of GCPS participants who were suitable and aware were not using PrEP, and 14% of PrEPARE participants who were PrEP-suitable and aware were unwilling to use it. PrEP use was lower among younger participants, bisexual and queer-identified participants compared with gay-identified participants, those outside metropolitan areas, and migrants without government-subsidised healthcare. Australia is among the few countries with repeated PrEP cascades that have been revised as implementation has evolved. We show that Australia has achieved near-universal PrEP awareness but highlight persistent inequities in uptake. Our findings demonstrate the value of long-term cascade monitoring and the need to address financial, geographic, and risk-perception barriers to close remaining gaps.

**Supplementary Information:**

The online version contains supplementary material available at https://doi.org/10.1007/s10461-026-05100-x.

## Resumen

Hemos monitoreado durante la última década el programa australiano de profilaxis preexposición (PrEP) frente al VIH, ofreciendo una perspectiva única sobre su implementación entre hombres gays, bisexuales y queer, y personas no binarias. Utilizando dos encuestas nacionales, los ‘GBQ+ Community Periodic Surveys’ (GCPS, 2014–2023, N=78.378) y el ‘PrEPARE Project’ (2017–2023, N=5.278), construimos y refinamos cascadas de PrEP para evaluar idoneidad, conocimiento, uso y vinculación con los servicios de salud. Para 2023, se observó un conocimiento casi universal de la PrEP entre las personas consideradas idóneas. El uso de PrEP aumentó de <1% a 30% en los GCPS y de 21% a 46% en PrEPARE. Sin embargo, persistieron desigualdades, con menor uso entre personas más jóvenes, participantes bisexuales y queer, quienes viven fuera de áreas metropolitanas y migrantes sin acceso a atención sanitaria subsidiada. Estos hallazgos destacan el valor del monitoreo longitudinal y la necesidad de intervenciones focalizadas.

## Introduction

HIV pre-exposure prophylaxis (PrEP) – the regular use of antiretroviral medications – is highly effective at preventing HIV [1–3]. In Australia, PrEP rollout has coincided with a large decline in annual HIV diagnoses [4]. Annual HIV diagnoses decreased nationally from 758 in 2016 to 456 in 2023 among men who have sex with men, representing 7.7 and 4.7 diagnoses per 100,000 population, respectively [5]. Governments have set ambitious targets to further increase PrEP coverage among those at highest risk of HIV acquisition, primarily gay, bisexual and queer men and non-binary people (GBQ + people). The Australian Government set targets to increase the use of one or more effective prevention strategies (including PrEP) among people at risk of HIV to 90% by 2025 and 95% by 2030 [6]. In view of these priorities, our work examines PrEP suitability over time, in line with Australian clinical guidelines [7], and identifies potential barriers to PrEP initiation using cascades developed from existing survey-based monitoring systems.

This article builds on two cascades we previously developed to monitor PrEP implementation among GBQ + people in Australia, which demonstrated steady increases in PrEP suitability, awareness, and use from 2014 to 2018 [8]. However, gaps remained, particularly between PrEP awareness and use, influenced by geographic location, socioeconomic status, and healthcare service and community engagement [8]. PrEP use was much more common among study participants in urban areas and those with higher education levels. More recent behavioral surveillance shows a diversification of PrEP use patterns, with a shift from daily use to on-demand (2–1-1) dosing [9], particularly since the COVID-19 epidemic began, noting that only oral PrEP was available in Australia at the time of writing. This shift has important implications for cascade monitoring, as non-daily users may engage with health services differently, including attending services less frequently for sexual health testing and monitoring. As these changes unfold and new PrEP modalities become available, it is essential that cascade models evolve to reflect the complexity of real-world PrEP use in Australia.

There has been growing global recognition of the need to monitor PrEP implementation systematically [10, 11]. PrEP cascades are one way to do this, assessing modifiable steps that may affect PrEP use and persistence, such as awareness of the intervention, willingness to use it, discussing PrEP with a provider, obtaining a prescription, adhering to dosing, and participating in monitoring or follow up visits [12, 13]. Several reviews have synthesized data on PrEP cascades across key populations affected by HIV, including GBQ + and transgender people, and racial and cultural minorities [14–16]. These reviews identified low uptake and suboptimal adherence as key challenges across cascade steps, even among individuals who are aware of and willing to use PrEP. Structural barriers such as healthcare access, provider availability, cost, and HIV-related stigma can significantly affect PrEP engagement [14, 16]. Racial and cultural disparities in cascade progression have also been observed, with minorities in the US, such as Black and Latino men who have sex with men facing additional barriers to healthcare engagement [14, 17, 18]. Importantly, international comparisons show variation in how cascades are constructed and measured, underscoring the importance of context-specific monitoring frameworks, for example, reflecting populations most at risk of HIV acquisition, the way health systems are organized and accessed, and local recommendations for dosing and monitoring [13, 19]. In other words, while PrEP cascades can highlight structural barriers that are common across different health systems, ongoing monitoring of contextual conditions is integral to support local goals, such as Australia’s ambitious prevention coverage targets [6].

PrEP was made available in Australia through demonstration projects from 2014 [8, 20]. It was subsequently listed in 2018 for public subsidy through Medicare, Australia’s government-subsidized healthcare scheme, and can now be prescribed by any doctor. Australian citizens and permanent residents have access to Medicare, which subsidises the cost of clinical consultations and PrEP (∼AU$25 for 30 pills, or AU$7.70 with a concession card), and fully subsidises the cost of laboratory testing [21]. Most short-term residents are ineligible for Medicare and instead face high out-of-pocket costs, particularly for clinical care. With a prescription, Medicare-ineligible individuals can purchase PrEP at full price from Australian pharmacies or legally import it from overseas at lower cost (∼AU$23 for 30 pills). Some jurisdictions also provide limited free or low-cost PrEP programs for those without Medicare. Equity in access is therefore a recognized concern, particularly for migrants [22, 23].

The aim of this article was to assess gaps in PrEP uptake by comparing two PrEP cascades in Australia. We refined two existing PrEP cascades using data from ongoing, national studies of GBQ + people; these permit evaluation of different points of intervention based on available behavioral measures in the two surveys, and assessment of whether similar progress has been made over time across the two cascades, including changes in retention between cascade steps and intervention points. For both cascades, we assessed changes over time in PrEP suitability, awareness, uptake, and routine monitoring (the four steps in common across the two cascades). Acknowledging that non-daily PrEP dosing has become much more common since COVID-19 [9], we used six-monthly testing as a marker of ongoing healthcare engagement rather than quarterly testing, which we had used in the original PrEPARE cascade [8]. For the PrEPARE cascade, we also distinguished steps for lifetime and current PrEP use to show PrEP discontinuation over time. After revising the cascades, we then identified the points at which suitable people were either retained in or dropped out of the cascade, in order to determine opportunities to increase PrEP coverage and persistence.

## Methods

### Participants and Procedures

Data were taken from two Australian national, repeated, cross-sectional studies: the GBQ + Community Periodic Surveys (hereafter, the GCPS), Australia’s primary HIV behavioral surveillance study, and the PrEPARE Project, a monitoring study of GBQ + people’s attitudes to and use of different HIV prevention methods. The methods for both studies have been described previously [24, 25]. Neither the GCPS nor PrEPARE participants were compensated financially.

The GCPS primarily engage GBQ + people in metropolitan areas, the population that continues to be most affected by HIV in Australia [6]. The GCPS are conducted in seven Australian states and territories. Annual recruitment occurs in the three most populous states, with biennial recruitment elsewhere. The surveys commenced in New South Wales in 1996, and in Victoria, Queensland, South Australia and Western Australia from 1998. The Australian Capital Territory was included from 2000 and Tasmania from 2014. Recruitment occurs primarily in-person at community venues and pride festival events, supplemented by online recruitment. Eligible participants were Australian residents, men (cisgender or transgender) who identified as gay or bisexual or had had sex with another man in the past five years, or non-binary people who had had sex with gay, bisexual or queer men in the past five years. Eligible participants needed to be aged ≥ 16 years old for online recruitment and ≥ 18 for face-to-face recruitment. Verbal informed consent was obtained from in-person participants, and online participants provided electronic informed consent.

PrEPARE is an online survey conducted biennially among GBQ + people in Australia since 2011. Recruitment is driven primarily by paid advertisements on Facebook and Instagram and the dating/hook up app Grindr. Potential participants were directed to the Qualtrics survey platform with information about the study. Participants provided consent before starting the survey. Eligible participants were aged ≥ 16 years old, did not identify as female, did not identify as heterosexual, and lived in Australia; that is, male or non-binary and gay, bisexual or queer-identified people could participate. Ineligible participants were screened out automatically if they did not meet any of the inclusion criteria. The studies were approved by the ethics committees of UNSW Sydney and the community organizations ACON and Thorne Harbour Health.

### Measures

Both surveys assess demographics, recent sexual behavior, HIV and sexual health testing, drug use, the use of different HIV prevention methods, while PrEPARE also assessed broader attitudes toward biomedical HIV prevention methods. The denominator for both cascades was all HIV-negative, untested, and unknown status participants in each sample; participants living with HIV were excluded. As this was a behavioural surveillance study, PrEP suitability was defined based on reported behavioural indicators of all participants who might benefit from PrEP, and HIV testing was treated as a subsequent cascade stage capturing engagement in care rather than a prerequisite for inclusion in the denominator.

The GCPS cascade consisted of the following steps:

1. Suitable for PrEP
2. Aware of PrEP
3. Used PrEP (in the last 6 months)
4. Tested for HIV (in the last 6 months)

Participants were considered in steps 2–4 if they met the conditions for the previous step(s). Suitability for PrEP was adapted from Australian prescribing guidelines [7] and operationalized as shown in Supplemental Table 1 across all years. Consistent with the Australian National PrEP Guidelines, we use the term *PrEP suitability* to refer to behavioral and contextual circumstances indicating likely benefit from PrEP, rather than *eligibility*, which in Australia is primarily used to denote clinical safety requirements.

Awareness of PrEP was assessed with the question, “Have you heard about pre-exposure prophylaxis (PrEP)?” with participants who answered “I’ve heard about it” classified as aware (step 2). Recent PrEP use (step 3) included affirmative responses to the question, “In the last 6 months, did you take PrEP to protect yourself from HIV?”, and included daily and non-daily oral PrEP. Participants were asked “When were you last tested for HIV”, and responses of up to six months were included in step 4.

The PrEPARE cascade consisted of the following steps:

1. Suitable for PrEP
2. Aware of PrEP
3. Willing to use PrEP
4. Discussed PrEP with a doctor
5. Ever used PrEP
6. Used PrEP (in the last 6 months)
7. Tested for HIV (in the last 6 months)
8. Increased psychosexual wellbeing from PrEP.

To be included in step 2 onward, participants had to meet the criteria for the preceding steps. Based on items available in the PrEPARE questionnaire, suitability for PrEP was operationalized as shown in Supplemental Table 1. Awareness of PrEP was assessed with the question, “How much have you heard about pre-exposure prophylaxis (PrEP)?” with participants who answered “A little” or “A lot” (vs. Never heard) classified as aware (step 2). Willingness to use PrEP was assessed with a previously validated 7-item scale (Cronbach’s alpha = 0.78–0.86) scored from 1 to 5 [26]. Participants were classified as willing (step 3) if their mean score was ≥ 4. Unlike the GCPS, PrEPARE distinguished between lifetime and current PrEP use. Participants who answered “Yes” to the question “Have you ever discussed pre-exposure prophylaxis (PrEP) with a doctor?” were included in step 4. Participants who answered “Yes” to the question “Have you ever taken PrEP (anti-HIV drugs BEFORE sex) to reduce the chance of HIV infection?” were included in step 5. Recent PrEP use (step 6) was assessed with the question, “In the last six months, did you take PrEP?” (Yes/No). Participants were excluded from step 6 if they indicated in a subsequent question on PrEP dosing that they had stopped taking PrEP permanently. As with the GCPS, daily and non-daily oral PrEP users were both included and PrEP dosing is reported separately to the cascades.

It was assumed that all PrEP users were aware of, willing to use, and had discussed PrEP with a doctor (they were not shown these questions). Only PrEP users who were classified as PrEP suitable were included in the cascade. PrEP users who reported they had been tested for HIV in the previous six months were included in step 7. PrEP users were classified as having increased psychosexual wellbeing (step 8) if their mean score was ≥ 4 on the 4-item *Sexual confidence and reduced HIV concern from PrEP* scale (Cronbach’s alpha = 0.78) scored from 1 to 5; the scale included items such as “Being on PrEP makes me feel confident about sex” and “I am less worried about getting HIV because of PrEP” [26].

### Analyses

Frequencies and percentages were computed for all variables, stratified by the relevant comparison group. Several analytic techniques were used to compare groups. First, logistic regression assessed similarities and differences in participant characteristics between the GCPS (0) and PrEPARE (1). Sample comparisons included the most recent, national data from each study so that all participating jurisdictions were included. For the most recent GCPS rounds (N = 9,177), six jurisdictions in which recruitment occurred between November 2022 (South Australia) and December 2023 (Western Australia) were included. For PrEPARE (N = 1,907), the most recent data were collected in June–July 2023.

Second, clustered bar charts for the two cascades were constructed showing each cascade step, stratified by year. The primary denominator was the total number of HIV-negative and untested/unknown status participants in each survey year. The secondary denominator was the number of participants retained in the previous step of the cascade (for step two onward). The implication is that gaps between steps indicate points of attrition in prevention engagement within the study samples, rather than independence between stages. The GCPS cascade included ten years of data (2014–2023), showing changes from the first year of formal PrEP implementation in Australia [20]. The PrEPARE cascade included four survey rounds (2017–2023); this period was chosen because some of the measures were not included in the survey prior to 2017 (e.g., psychosexual wellbeing).

Third, trends over time were assessed for the two cascades; generalized linear models assessed changes in each cascade step and each PrEP suitability criterion. The dependent variable was the outcome of interest in binary format, with the survey year and other covariates as independent variables to control for changes in sampling over time. For the GCPS and PrEPARE, common covariates were age, sexual identity, employment, state/territory of residence, region of residence (inner metro vs. outer metro vs. rural/remote), and country of birth (born in Australia vs. overseas); these were used as control variables in the trend analyses. Income was not measured in the GCPS but was used as a covariate in PrEPARE. Generalized linear models assume independent observations, in which each response is derived from a distinct, unrelated individual. While it was not possible to determine whether some participants may have completed the surveys in multiple years between 2014 and 2023, the datasets did not include repeated measures or matched cases. We therefore determined that it was methodologically appropriate to assess trends using generalized linear models.

Fourth, break point analyses were conducted for each cascade (assessing the biggest drop between cascade steps by absolute proportion that appeared modifiable to improve PrEP uptake). Break point analyses included 2023 data only, consistent with the cascade stratifications. Crude drop-offs between cascade steps were calculated by subtracting the number of individuals retained at each step from those at the preceding step. The difference was then calculated as a percentage of the previous step’s total. Multivariable logistic regression (GCPS) and multinomial logistic regression (PrEPARE) compared participants between the two steps that had the greatest proportional loss. For PrEPARE, most participants were assumed to be ‘willing’ to use PrEP based on prior PrEP use, so we separated participants into three categories (1) unwilling, (2) willing but never used PrEP, and (3) ever used PrEP. Given this classification, and the small group size of 80 participants in category 2, independent variables were selected carefully to minimize the number of predictors to preserve statistical power. Independent variables were recoded to binary format where logical. Two HIV and STI testing variables were excluded due to strong collinearity with PrEP use, as well as condomless anal intercourse with regular partners.

For all regression models, variables that were significantly associated at a bivariable level (*p* < 0.05) were entered simultaneously into multivariable models in a single block. We report adjusted odds ratios, 95% confidence intervals, and *p*-values. Analyses were conducted using Stata version 17.0 (StataCorp, College Station, TX).

## Results

### Participant Characteristics

Between 2014 and 2023, 78,378 HIV-negative and untested/unknown status participants completed the GCPS. The median age was 34 years old (interquartile range (IQR) = 27–44), 70.2% were born in Australia, 53.5% resided in inner-metropolitan areas, 54.8% had a university degree, and 65.3% were full-time employed. Most participants resided in New South Wales (34.0%), Victoria (31.3%), or Queensland (20.2%), with 14.5% living in other states or territories. Most identified as gay (85.3%). Gender identity and sex assigned at birth were collected from 2017. Between 2017 and 2023, participation by trans and gender diverse people in the GCPS increased: from 1.4% to 2.3% for trans men, and from 0.5% to 3.8% for non-binary people. Cisgender men comprised 97.0% of the sample in 2017 and 92.7% in 2023.

Between 2017 and 2023, 5,278 HIV-negative and untested/unknown status participants completed the PrEPARE survey. The median age was 35 years old (IQR = 27–46). 70.2% were born in Australia, 53.5% resided in inner-metropolitan areas, 54.8% had a university degree, and 65.3% were employed full time. Most participants resided in New South Wales (34.0%), Victoria (27.8%), or Queensland (17.5%), and 20.7% resided in other states or territories. Most identified as gay (85.3%). Most participants were cisgender men (96.5%), 2.0% were transgender men and 1.5% were non-binary.

Supplemental Table 2 compares participant characteristics between the GCPS and PrEPARE in the most recent rounds (2022–23) using multivariable logistic regression and variables common to both studies. This analysis was performed acknowledging that the two studies hold common eligibility criteria but different recruitment methods, which may result in different sample characteristics. PrEPARE participants were more likely than GCPS participants to be younger; that is, less likely to be aged 26–35 (*aOR* = 0.50, 95%CI: 0.43, 0.59, *p* < 0.001), 36–55 (*aOR* = 0.60, 95%CI: 0.52, 0.70, *p* < 0.001), or ≥ 56 years (*aOR* = 0.47, 95%CI: 0.38, 0.57, *p* < 0.001) relative to ≤ 25. They were more likely to reside outside New South Wales, specifically in Victoria (*aOR* = 1.15, 95%CI: 1.01, 1.32, *p* = 0.035) and in the Australian Capital Territory/Northern Territory/Tasmania (*aOR* = 2.67, 95%CI: 2.10, 3.40, *p* < 0.001), and less likely to reside in South Australia (*aOR* = 0.78, 95%CI: 0.63, 0.97, *p* = 0.025) or Western Australia (*aOR* = 0.79, 95%CI: 0.64, 0.97, *p* = 0.026). They were also more likely to live in outer metropolitan (*aOR* = 1.40, 95%CI: 1.23, 1.59, *p* < 0.001) or regional/remote areas versus inner-metropolitan (*aOR* = 1.69, 95%CI: 1.45, 1.97, *p* < 0.001), to have a university degree (*aOR* = 1.23, 95%CI: 1.09, 1.37, *p* < 0.001), to have had a recent STI test (*aOR* = 3.45, 95%CI: 2.93, 4.05, *p* < 0.001), and to report recent condomless anal intercourse with casual partners (*aOR* = 1.39, 95%CI: 1.23, 1.58, *p* < 0.001). In addition, PrEPARE participants were less likely than GCPS participants to identify as Aboriginal and/or Torres Strait Islander (*aOR* = 0.61, 95%CI: 0.43, 0.87, *p* = 0.007), to have had more than ten male partners in the last six months (*aOR* = 0.83, 95%CI: 0.73, 0.95, *p* = 0.008), and to have been recently tested for HIV (*aOR* = 0.77, 95%CI: 0.67, 0.89, *p* < 0.001). No significant differences were observed in employment, sexual identity, condom use with regular partners, or recent PrEP use after adjustment.

### PrEP Cascade, GCPS

**Fig. 1:**
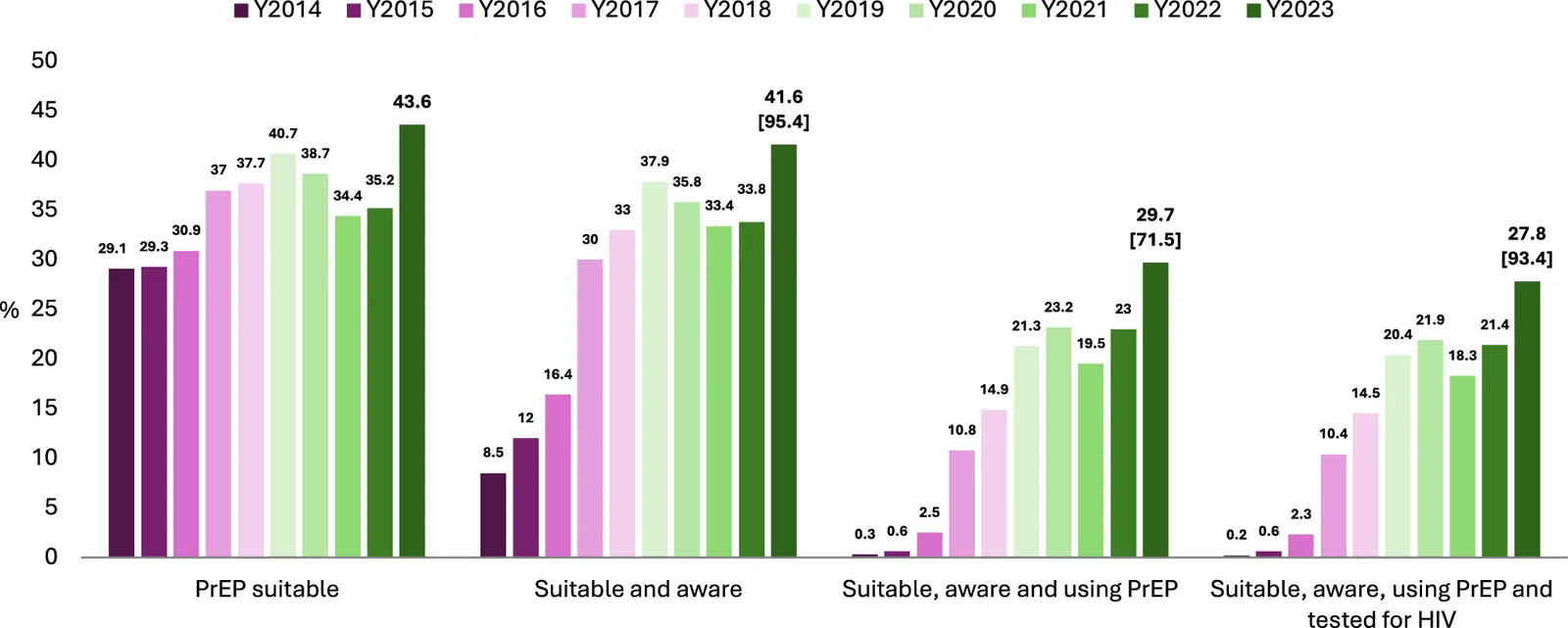
PrEP cascade for HIV-negative and untested GBQ + people in the GCPS, 2014–2023. Percentages in parentheses are calculated with the previous cascade step as the denominator

Figure 1 shows the cascade derived from the GCPS. There were between 6,413 and 9,001 HIV-negative and untested/unknown status participants each year. Between 2014 and 2023, PrEP suitability increased from 29.1% (1,958/6,731) to 43.6% (3,647/8,365), with most classified as suitable based on receptive condomless anal intercourse with a casual male partner (see Supplemental Table 3 for trends in PrEP suitability). Between 2014 and 2023, awareness increased from 8.5% (574/6,731) to 41.6% (3,480/8,365), PrEP use increased from 0.3% (19/6,731) to 29.7% (2,487/8,365), and recent HIV testing increased from 0.2% (16/6,731) to 27.8% (2,323/8,365). All cascade steps showed significant upward trends over time (all *p* < 0.001; see Supplemental Table 4 for adjusted odds ratios and 95% confidence intervals), controlling for covariates.

Between 2014 and 2023, PrEP awareness among PrEP-suitable participants increased from 29.3% (574/1,958) to 95.4% (3,480/3,647). PrEP use among participants who met steps 1–2 increased from 3.3% (19/574) to 71.5% (2,487/3,480); that is, 28.5% of PrEP-suitable and aware participants were not using PrEP in 2023. Recent HIV testing among PrEP users who met steps 1–3 increased from 84.2% (16/19) to 93.4% (2,323/2,487) over the same period. Non-daily dosing was measured from 2019. Between 2019 and 2023, daily PrEP use decreased from 93.0% to 68.8% while on-demand use increased from 7.0% to 26.3%.

Between 2014 and 2016, the largest crude drop-off was between PrEP suitability and awareness (steps 1 and 2). From 2017 the largest crude drop-off was between awareness and use (steps 2 and 3). However, between 2014 and 2023, the greatest proportional drop-off, relative to the previous step, was between steps 2 and 3 (awareness and use), although this decreased from 96.7% in 2014 to 28.5% in 2023.

The break point analysis is shown in Table 1. Multivariable logistic regression compared PrEP suitable and aware participants who were and were not using PrEP. Adjusting for covariates, PrEP users had greater odds of being aged 26–35 (*aOR* = 1.52, 95%CI: 1.12, 2.08, *p* = 0.007), 36–55 (*aOR* = 1.74, 95%CI: 1.26, 2.41, *p* = 0.001), or ≥ 56 years (*aOR* = 2.54, 95%CI: 1.63, 3.95, *p* < 0.001), compared to those aged ≤ 25. They were also more likely to be university educated (*aOR* = 1.43, 95%CI: 1.16, 1.76, *p* = 0.001), identify as gay versus bisexual, queer or another identity (*aOR* = 1.83, 95%CI: 1.40, 2.38, *p* < 0.001), meet sex partners via mobile apps (*aOR* = 1.68, 95%CI: 1.31, 2.15, *p* < 0.001) or at saunas or sex-on-premises venues (*aOR* = 1.35, 95%CI: 1.09, 1.69, *p* = 0.007), report more than 10 male partners in the last six months (*aOR* = 1.73, 95%CI: 1.36, 2.21, *p* < 0.001), report any condomless anal intercourse with casual partners (*aOR* = 1.26, 95%CI: 1.02, 1.55, *p* = 0.033) and with regular partners (*aOR* = 2.24, 95%CI: 1.70, 2.96, *p* < 0.001) in the last six months. In relation to testing, PrEP users were more likely to report recent HIV testing in the last six months (*aOR* = 3.32, 95%CI: 2.56, 4.31, *p* < 0.001), more than two HIV tests in the last 12 months (*aOR* = 4.21, 95%CI: 3.39, 5.22, *p* < 0.001), and any STI test in the last 12 months (*aOR* = 2.54, 95%CI: 1.78, 3.63, *p* < 0.001). They were also more likely to report the use of amyl nitrites (‘poppers’) (*aOR* = 1.37, 95%CI: 1.10, 1.71, *p* = 0.004) and erectile dysfunction medication (*aOR* = 1.30, 95%CI: 1.03, 1.65, *p* = 0.029). PrEP users were less likely to live in outer-metropolitan areas versus inner-metropolitan (*aOR* = 0.74, 95%CI: 0.58, 0.95, *p* = 0.018).

**Table 1:** Comparison of eligible and aware participants using and not using PrEP, GCPS, 2023

	Eligible, aware and not using PrEP (n = 933)	Eligible, aware and using PrEP (n = 2,487)	OR (95% CI)	*p* value	AOR (95% CI)	*p* value
Age						
25 years and under	194 (19.5)	239 (9.6)	Ref		Ref	
26–35	363 (36.6)	1,034 (41.6)	2.31 (1.85–2.89)	< .001	1.52 (1.12–2.08)	.007
36–55	339 (34.1)	978 (39.3)	2.34 (1.87–2.94)	< .001	1.74 (1.26–2.41)	.001
56 years and over	97 (9.8)	236 (9.5)	1.97 (1.46–2.67)	< .001	2.54 (1.63–3.95)	< .001
State/territory						
New South Wales	372 (37.5)	1,042 (41.9)	Ref		Ref	
Victoria	242 (24.4)	791 (31.8)	1.17 (0.97–1.41)	.105	1.01 (0.79–1.29)	.936
Queensland	217 (21.9)	421 (16.9)	0.69 (0.57–0.85)	< .001	0.94 (0.71–1.24)	.644
Western Australia	117 (11.8)	194 (7.8)	0.59 (0.46–0.77)	< .001	0.86 (0.60–1.23)	.403
Tasmania	45 (4.5)	39 (1.6)	0.31 (0.20–0.48)	< .001	0.86 (0.47–1.58)	.628
Residential location						
Inner metropolitan	492 (49.5)	1,608 (64.7)	Ref		Ref	
Outer metropolitan	280 (28.2)	535 (21.5)	0.58 (0.49–0.70)	< .001	0.74 (0.58–0.95)	.018
Regional and remote	182 (18.3)	271 (10.9)	0.46 (0.37–0.56)	< .001	0.80 (0.59–1.09)	.164
Postcode not provided or unmatched	39 (3.9)	73 (2.9)	0.57 (0.38–0.86)	.007	0.68 (0.40–1.16)	.155
Born in Australia	306 (30.8)	895 (36.0)	1.26 (1.08–1.48)	.004	1.03 (0.83–1.27)	.802
Anglo- or Celtic-Australian ethnicity	643 (64.8)	1,576 (63.4)	0.94 (0.81–1.10)	.443		
Aboriginal and/or Torres Strait Islander	35 (3.5)	64 (2.6)	0.72 (0.48–1.10)	.129		
University degree	523 (52.8)	1,717 (69.2)	2.01 (1.73–2.34)	< .001	1.43 (1.16–1.76)	.001
Full-time employed	687 (69.2)	1,945 (78.2)	1.60 (1.36–1.89)	< .001	1.13 (0.89–1.43)	.309
Socially engaged with GBQ + people (vs. not)^1^	640 (64.5)	1,959 (78.8)	2.05 (1.74–2.41)	< .001	1.05 (0.84–1.32)	.654
Gay-identified	758 (76.7)	2,188 (88.3)	2.29 (1.89–2.77)	< .001	1.83 (1.40–2.38)	< .001
How men met their male partners^2^						
Internet	419 (42.2)	1,258 (50.6)	1.40 (1.21–1.63)	< .001	0.98 (0.79–1.21)	.827
Mobile app	628 (63.2)	2,129 (85.6)	3.46 (2.91–4.10)	< .001	1.68 (1.31–2.15)	< .001
Gay bar	285 (28.7)	1,155 (46.4)	2.15 (1.84–2.52)	< .001	1.06 (0.85–1.33)	.584
Saunas or sex venues	270 (27.2)	1,283 (51.6)	2.85 (2.43–3.35)	< .001	1.35 (1.09–1.69)	.007
More than 10 male partners^2^	160 (16.2)	1,213 (48.9)	4.95 (4.10–5.96)	< .001	1.73 (1.36–2.21)	< .001
Any condomless sex with regular partners^2^	710 (71.5)	2,214 (89.0)	3.23 (2.68–3.90)	< .001	2.24 (1.70–2.96)	< .001
Any condomless sex with casual partners^2^	619 (62.3)	1,818 (73.1)	1.64 (1.40–1.92)	< .001	1.26 (1.02–1.55)	.033
Any group sex^2^	351 (35.3)	1,502 (60.4)	2.79 (2.39–3.25)	< .001	1.07 (0.85–1.33)	.575
Recent HIV test^2^	513 (51.7)	2,323 (93.4)	13.25 (10.84–16.21)	< .001	3.32 (2.56–4.31)	< .001
Had more than two HIV tests^3^	208 (20.9)	1,839 (73.9)	10.71 (8.97–12.79)	< .001	4.21 (3.39–5.22)	< .001
Any STI test^3^	702 (70.7)	2,386 (95.9)	9.79 (7.69–12.47)	< .001	2.54 (1.78–3.63)	< .001
Any STI diagnosis^3^	341 (34.3)	1,395 (56.1)	2.44 (2.10–2.85)	< .001	1.13 (0.90–1.40)	.291
Used party drugs for sex^2^	916 (92.2)	2,358 (94.8)	1.54 (1.15–2.06)	.004	0.78 (0.51–1.21)	.273
Any injecting drug use^2^	50 (5.0)	98 (3.9)	0.77 (0.55–1.10)	.149		
Drugs used^2^						
Amyl nitrites (‘poppers’)	538 (54.2)	1,796 (72.2)	2.20 (1.89–2.56)	< .001	1.37 (1.10–1.71)	.004
Cannabis	406 (40.9)	1,026 (41.3)	1.02 (0.87–1.18)	.842		
Crystal methamphetamine	141 (14.2)	287 (11.5)	0.79 (0.63–0.98)	.031	0.75 (0.53–1.07)	.11
Ecstasy	251 (25.3)	823 (33.1)	1.46 (1.24–1.73)	< .001	1.14 (0.87–1.49)	.344
Erectile dysfunction medication	250 (25.2)	1,078 (43.3)	2.27 (1.93–2.68)	< .001	1.30 (1.03–1.65)	.029
Gamma hydroxybutyrate (GHB)	142 (14.3)	438 (17.6)	1.28 (1.04–1.57)	.018	1.11 (0.78–1.57)	.57
Ketamine	172 (17.3)	621 (25.0)	1.59 (1.32–1.92)	< .001	1.18 (0.87–1.60)	.284

Data are n(%) unless otherwise stated. PrEP = pre-exposure prophylaxis, OR = odds ratio, CI = confidence interval, SD = standard deviation, STI = sexually transmissible infection, PEP = post-exposure prophylaxis

^1^Defined as participants who reported that most or all of their friends were GBQ + people and that they spent some or a lot of their free time with GBQ + people

^2^In the last six months

^3^In the last 12 months

### PrEP Cascade, PrEPARE

**Fig. 2:**
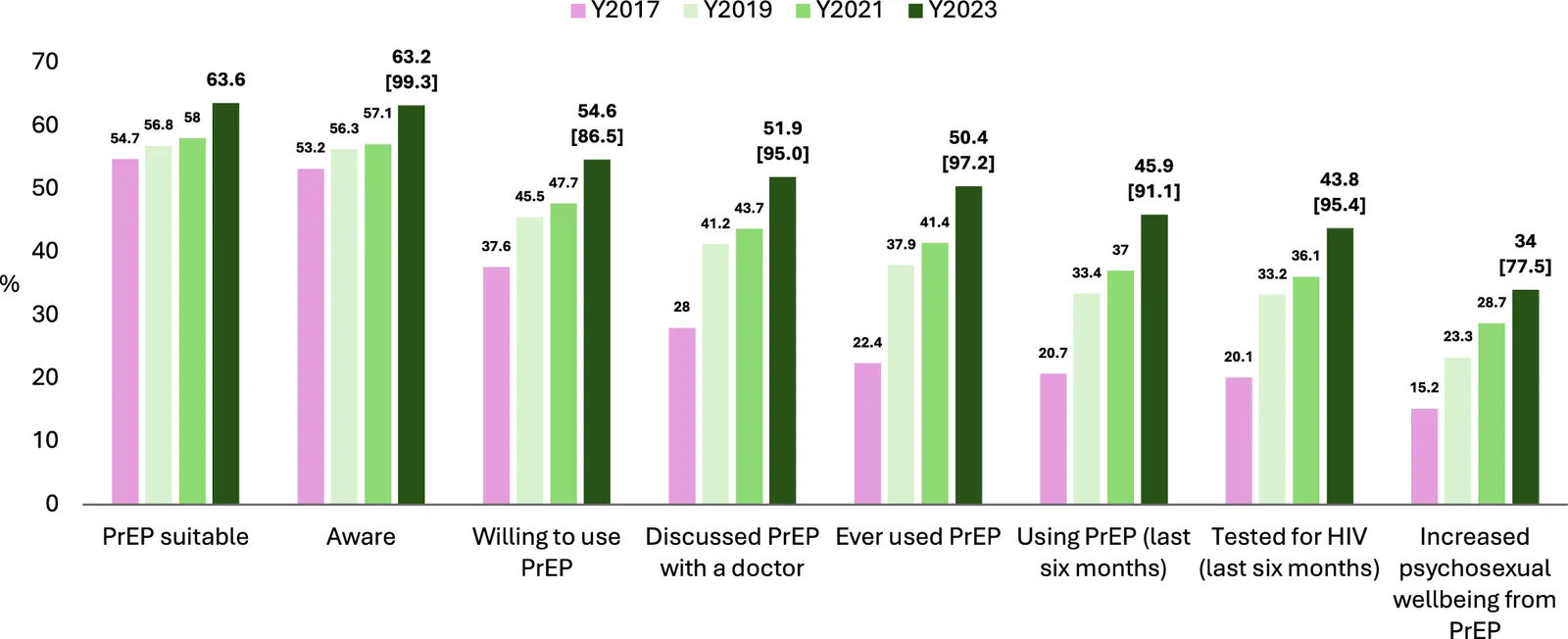
PrEP cascade for HIV-negative and untested GBQ + people in the PrEPARE Project, 2017–2023. Percentages in parentheses are calculated with the previous cascade step as the denominator

Figure 2 shows the PrEP cascade from PrEPARE. There were between 1,038 and 1,907 HIV-negative and untested/unknown status participants each year. Between 2017 and 2023, PrEP suitability increased from 54.7% (568/1,038) to 63.6% (1,213/1,907), with most classified as suitable based on having any condomless anal intercourse with a casual male partner (see Supplemental Table 5 for trends in PrEP suitability). Between 2017 and 2023, awareness increased from 53.2% (552/1,038) to 63.2% (1,205/1,907), and willingness to use PrEP increased from 37.6% (390/1,038) to 54.6% (1,042/1,907). Over the same period, the proportion who discussed PrEP with a doctor increased from 28.0% (291/1,038) to 51.9% (990/1,907), and those who had ever used PrEP increased from 22.4% (232/1,038) to 50.4% (962/1,907). Current PrEP use increased from 20.7% (215/1,038) to 45.9% (876/1,907), and recent HIV testing within the past six months increased from 20.1% (209/1,038) to 43.8% (836/1,907). The proportion reporting increased psychosexual wellbeing from PrEP increased from 15.2% (158/1,038) to 34.0% (648/1,907). All cascade steps showed significant upward trends over time (all *p* < 0.001; see Supplemental Table 6 for adjusted odds ratios and 95% confidence intervals), controlling for covariates.

Between 2017 and 2023, PrEP awareness among PrEP-suitable participants increased from 97.2% (552/568) to 99.3% (1,205/1,213). Willingness to use PrEP among those who were aware increased from 70.7% (390/552) to 86.5% (1,042/1,205). Discussing PrEP with a doctor among those willing to use PrEP increased from 74.6% (291/390) to 95.0% (990/1,042). Ever using PrEP among those who had discussed it with a doctor increased from 79.7% (232/291) to 97.2% (962/990). Current PrEP use among those who had ever used it decreased from 92.7% (215/232) to 91.1% (876/962), and HIV testing among current PrEP users decreased from 97.2% (209/215) to 95.4% (836/876). The proportion of PrEP users who had recently tested and reported increased psychosexual wellbeing rose from 75.6% (158/209) to 77.5% (648/836) over the same period. Between 2017 and 2023, daily PrEP use decreased from 98.1% to 65.2% while on-demand use increased from 0.5% to 30.7%.

In 2017, the largest crude drop-off in the PrEPARE cascade was between awareness and willingness to use PrEP (steps 2 and 3). The gap between awareness and willingness decreased in each round, from 29.3% in 2017 to 13.5% in 2023. The next largest gap has been between recent testing for HIV and increased psychosexual wellbeing from PrEP (steps 7 and 8). The relative drop-off between testing and psychosexual wellbeing has fluctuated from 24.4% in 2017 to 29.9% in 2019, and declined to 22.5% in 2023. Although the absolute and relative percentage differences were larger between steps 7 and 8 in 2023, we have focused on the earlier intervention point in the cascade to understand the characteristics of participants who were suitable and aware of PrEP but unwilling to use it.

The break point analysis is shown in Table 2. Multinomial logistic regressions compared participants who were PrEP users (past or present), participants who had never used PrEP but were willing to do so, and participants who never used PrEP and were unwilling. First, no variables were independently associated with willingness to use PrEP relative to unwillingness. Second, compared to the unwilling group, prior PrEP users had greater odds of having access to government-subsidized healthcare (*aOR* = 2.94, 95%CI: 1.60, 5.40, *p* = 0.001), identifying as gay versus bisexual, queer or another identity (*aOR* = 2.65, 95%CI: 1.71, 4.13, *p* < 0.001), reporting more than ten male partners in the last six months (*aOR* = 2.44, 95%CI: 1.46, 4.09, *p* = 0.001), reporting any condomless anal intercourse with casual partners in the last six months (*aOR* = 4.95, 95%CI: 2.82, 8.68, *p* < 0.001), reporting any STI diagnosis (*aOR* = 4.86, 95%CI: 2.91, 8.11, *p* < 0.001), reporting recent PEP use (*aOR* = 3.12, 95%CI: 1.42, 6.87, *p* = 0.005), perceiving themselves as at lower risk of acquiring HIV (unlikely/neutral vs. likely; *aOR* = 5.78, 95%CI: 2.36, 14.11, *p* < 0.001), knowing at least one person taking PrEP (*aOR* = 2.24, 95%CI: 1.39, 3.63, *p* = 0.001), and knowing at least one person living with HIV (*aOR* = 1.65, 95%CI: 1.11, 3.63, *p* = 0.013). Prior PrEP users had lower odds of living in regional or remote areas versus inner-metropolitan areas (*aOR* = 0.46, 95%CI: 0.28, 0.76, *p* = 0.002). Third, compared to the willing group who had never used PrEP, prior PrEP users had greater odds of being aged ≥ 30 years (*aOR* = 2.08, 95%CI: 1.22, 3.54, *p* = 0.007), reporting any condomless anal intercourse with casual partners in the last six months (*aOR* = 5.64, 95%CI: 2.88, 11.05, *p* < 0.001), reporting any STI diagnosis (*aOR* = 3.30, 95%CI: 1.78, 6.14, *p* < 0.001), perceiving themselves to be at lower risk of acquiring HIV (*aOR* = 7.05, 95%CI: 2.53, 19.66, *p* < 0.001), and knowing at least one person living with HIV (*aOR* = 1.85, 95%CI: 1.10, 3.12, *p* = 0.021). Prior PrEP users had lower odds of living in regional/remote areas versus inner-metropolitan areas (*aOR* = 0.42, 95%CI: 0.22, 0.77, *p* = 0.005).

**Table 2:** Comparison of eligible and aware participants willing and unwilling to use PrEP, PrEPARE Project, 2023

	Cross-tabulations n (%)				Bivariable associations			Multinomial logistic regression					
	Total	Unwilling	Willing, never used PrEP	Used PrEP			Willing, never used PrEP (vs. unwilling)			Used PrEP (vs. unwilling)		Used PrEP (vs. willing, never used PrEP)	
	N = 1,205	n = 163	n = 80	n = 962	Chi-square	p value	aOR (95% CI)		p value	aOR (95% CI)	p value	aOR (95% CI)	p value
Age					27.9	< .001							
Under 30 years	358 (29.7)	64 (39.3)	40 (50.0)	254 (26.4)			Ref			Ref		Ref	
30 years and over	847 (70.3)	99 (60.7)	40 (50.0)	708 (73.6)			0.70 (0.39–1.28)		.248	1.46 (0.96–2.24)	.08	2.08 (1.22–3.54)	.007
State/territory					9.2	.16							
New South Wales	396 (32.9)	50 (30.7)	28 (35.0)	318 (33.1)									
Victoria	347 (28.8)	38 (23.3)	21 (26.2)	288 (29.9)									
Queensland	222 (18.4)	41 (25.2)	18 (22.5)	163 (16.9)									
Others	240 (19.9)	34 (20.9)	13 (16.2)	193 (20.1)									
Residential location					*	< .001							
Inner metropolitan	632 (52.4)	66 (40.5)	34 (42.5)	532 (55.3)			Ref			Ref		Ref	
Outer metropolitan	328 (27.2)	49 (30.1)	20 (25.0)	259 (26.9)			0.72 (0.36–1.43)		.351	0.66 (0.42–1.03)	.07	0.91 (0.49–1.69)	.776
Regional and remote	221 (18.3)	45 (27.6)	26 (32.5)	150 (15.6)			1.11 (0.56–2.22)		.757	0.46 (0.28–0.76)	.002	0.42 (0.22–0.77)	.005
Postcode not provided or unmatched	24 (2.0)	3 (1.8)	0 (0.0)	21 (2.2)			**						
Born in Australia	871 (72.3)	118 (72.4)	56 (70.0)	697 (72.5)	0.2	.89							
Aboriginal and/or Torres Strait Islander	20 (1.7)	4 (2.5)	2 (2.5)	14 (1.5)	1.2	.54							
University degree	795 (66.0)	95 (58.3)	39 (48.8)	661 (68.7)	18.1	< .001	0.73 (0.40–1.31)		.286	1.13 (0.75–1.71)	.551	1.56 (0.93–2.63)	.094
Full-time employed	897 (74.4)	109 (66.9)	47 (58.8)	741 (77.0)	18.6	< .001	0.68 (0.37–1.24)		.204	1.02 (0.66–1.57)	.938	1.50 (0.88–2.57)	.136
Government-subsidized healthcare (Medicare)	1,113 (92.4)	140 (85.9)	72 (90.0)	901 (93.7)	12.6	.002	1.87 (0.75–4.65)		.179	2.94 (1.60–5.40)	.001	1.57 (0.67–3.71)	.301
Gay-identified (vs. bi + , queer, other)	1,010 (83.8)	114 (69.9)	60 (75.0)	836 (86.9)	34.5	< .001	1.53 (0.82–2.86)		.185	2.65 (1.71–4.13)	< .001	1.74 (0.96–3.14)	.067
More than 10 male partners^1^	422 (35.0)	25 (15.3)	15 (18.8)	382 (39.7)	46.3	< .001	1.44 (0.67–3.05)		.348	2.44 (1.46–4.09)	.001	1.70 (0.90–3.23)	.104
Any condomless sex with casual partners^1^	1,078 (89.5)	128 (78.5)	59 (73.8)	891 (92.6)	51.8	< .001	0.88 (0.43–1.78)		.718	4.95 (2.82–8.68)	< .001	5.64 (2.88–11.05)	< .001
Any condomless sex with regular partners^1^	907 (75.3)	100 (61.3)	48 (60.0)	759 (78.9)	33.8	< .001	***						
Recent HIV test^2^	999 (82.9)	70 (42.9)	38 (47.5)	891 (92.6)	318.5	< .001	†						
Ever tested for STIs	1,106 (91.8)	125 (76.7)	54 (67.5)	927 (96.4)	138.6	< .001	†						
Any STI diagnosis^2^	502 (41.7)	28 (17.2)	20 (25.0)	454 (47.2)	61.5	< .001	1.47 (0.71–3.03)		.295	4.86 (2.91–8.11)	< .001	3.30 (1.78–6.14)	< .001
Recent PEP use^1^	137 (11.4)	8 (4.9)	7 (8.8)	122 (12.7)	8.9	.011	1.74 (0.60–5.06)		.311	3.12 (1.42–6.87)	.005	1.79 (0.76–4.25)	.183
Perceived likelihood of acquiring HIV					24.1	< .001							
Unlikely/neutral	1,171 (97.2)	152 (93.3)	73 (91.2)	946 (98.3)			0.82 (0.29–2.32)		.709	5.78 (2.36–14.11)	< .001	7.05 (2.53–19.66)	< .001
Likely	34 (2.8)	11 (6.7)	7 (8.8)	16 (1.7)			Ref			Ref		Ref	
Knows at least one person taking PrEP	1,057 (87.7)	119 (73.0)	60 (75.0)	878 (91.3)	56.0	< .001	1.22 (0.63–2.36)		.55	2.24 (1.39–3.63)	.001	1.84 (0.98–3.44)	.057
Knows at least one person living with HIV	709 (58.8)	65 (39.9)	30 (37.5)	614 (63.8)	49.1	< .001	0.89 (0.50–1.60)		.697	1.65 (1.11–2.44)	.013	1.85 (1.10–3.12)	.021
Drug enhanced sex^1^	287 (23.8)	37 (22.7)	20 (25.0)	230 (23.9)	0.2	.92							

PrEP = pre-exposure prophylaxis, aOR = adjusted odds ratio, CI = confidence interval, SD = standard deviation, STI = sexually transmissible infection, PEP = post-exposure prophylaxis

^1^In the last six months

^2^In the last 12 months

*Fisher’s exact test

**Omitted due to empty cells

***Omitted for parsimony, and to reduce events per variable

^†^Omitted due to strong association with PrEP use

## Discussion

We constructed two PrEP cascades using national Australian data to assess progress in PrEP rollout among GBQ + people. Internationally, our PrEP cascades are unique in showing the maturation of PrEP rollout over a ten-year period, the narrowing of gaps in many cascade steps, and a reduction in some disparities in PrEP use that we previously identified [8]. For example, we have observed consistent increases over time in PrEP suitability, PrEP awareness, use, and recent HIV testing across both cascades, despite the risk that PrEP use could plateau without new interventions to address disparities in access or the availability of novel PrEP modalities such as long-acting injections in Australia [12].

In 2023, nearly all PrEP-suitable participants were aware of PrEP. Although this figure is higher than a pooled global estimate of 50%, our findings are generally consistent with research indicating greater awareness over time and among high-income countries [27]. Despite large increases in PrEP awareness over time, we found that 28.5% of GCPS participants who were PrEP suitable and aware were not using PrEP, and 13.5% of PrEPARE participants who were PrEP suitable and aware were unwilling to use PrEP. Although community knowledge of PrEP is now widespread, the cascades show that there are persistent gaps between PrEP awareness and use, and awareness and willingness to use PrEP, which we discuss in turn.

The GCPS cascade provides a robust, population-level overview of PrEP uptake by GBQ + people over a decade in Australia. In the early years, the largest crude gap was between PrEP suitability and awareness, but as awareness has increased substantially, uptake or use has become the main point of attrition. In our breakpoint analysis of PrEP suitable and aware participants in the GCPS who were not using PrEP compared to PrEP users, we identified disparities by sexuality, age, education and residential location, which suggest that although PrEP access has broadened, it remains unevenly distributed; for example, participants who met suitability criteria and were aware of PrEP but were not using it were more likely to identify as bi + , queer, or another identity (compared with gay); were more likely to be aged under 25 years old and live in a suburban area; and were less likely to be university educated. This is consistent with analyses of disparities in PrEP uptake that we have identified before [28]. That said, we previously reported lower levels of PrEP use among non-Anglo-Australians and people without full-time work in 2017/18 [8], but in this analysis those gaps appear to have reduced. These results suggest that the various low-cost or free PrEP access schemes, particularly those targeting immigrants to Australia, have been successful in encouraging PrEP initiation among some PrEP-suitable people, in addition to the effect of PrEP’s public subsidy from 2018. In 2025, the Australian Government committed to funding PrEP for Medicare-ineligible residents (excluding short stay visitors) [29]. Continued monitoring of PrEP cascades will help track equity in PrEP access for this group.

The PrEPARE cascade adds additional depth to our PrEP monitoring by measuring additional steps between awareness and uptake, including willingness to use PrEP and having discussions with healthcare providers. Despite nearly universal PrEP awareness and sustained increases in willingness to use PrEP, there remains a small minority of participants who are unwilling to use PrEP. In the current study, nearly 90% of PrEP-suitable and aware participants were willing to use PrEP, which is higher than a global pooled estimate of 58% [27]; though it should be noted that disparate measures of willingness and acceptability, as well as differing denominators, make crude comparisons across settings unreliable. In our breakpoint analysis of PrEP-suitable and aware participants, no variables independently distinguished participants who were unwilling to use PrEP from those who were willing to use it. This result likely reflects a departure from other settings in which willingness has been shown to be more closely patterned alongside characteristics that align with structural barriers to PrEP access, including age, education and residential location [27, 30]. Our results instead suggest a need for broad-based interventions that better understand reservations in relation to PrEP, such as regular pill-taking or side effects, and the potential for novel PrEP modalities to address these concerns, rather than narrowly focusing on specific subgroups. However, when we further examined participants who were willing but had never used PrEP (compared with PrEP users), we found that willing non-users were generally younger, lived in regional areas, had fewer sex partners, and were less likely to report recent STI diagnoses, among other factors. These results are consistent with the GCPS cascade and prior research [28] in identifying age and residential location as sociodemographic disparities in uptake, however the PrEPARE cascade extends these findings by demonstrating that sociodemographic disparities are more strongly concentrated at the point of PrEP uptake rather than willingness. The results are also consistent with previous research that suggests GBQ + people may be less likely to start PrEP if they perceive themselves to be at less risk, for example, due to having fewer sexual partners [27, 31].

Consistent with our previous analysis of 2019 data [23], participants who were unwilling to use PrEP were less likely to have access to government-subsidized healthcare (Medicare), compared with PrEP users, thereby making PrEP more costly. Lack of Medicare coverage is associated with being a recent or temporary migrant in Australia, so this finding may point to migrants preferring to use other prevention methods (like condoms) [22], the cost of accessing PrEP without Medicare being a deterrent to its use in Australia [23, 28], or the difficulties migrants experience navigating the healthcare system due to language and other cultural barriers [32]. As with the GCPS cascade, the PrEPARE analysis showed that PrEP use was concentrated among participants with greater healthcare access in urban areas, which reaffirms that ongoing action is required to increase ease of access in suburban and regional locations [33]. Although PrEP users reported more partners and higher levels of condomless casual sex, all of the participants included in the prior cascade steps were deemed PrEP suitable (i.e., they reported some behavior associated with a risk of HIV acquisition). Previous work has found self-perceived low risk or infrequent HIV risk affects PrEP uptake or persistence [34]. Further research should also consider whether GBQ + people who are classified as ‘at-risk’ of HIV according to clinical guidelines recognize themselves as being ‘at-risk’, and in turn, the extent to which risk-avoidance messaging motivates users compared to empowerment and self-care. For example, willingness to take PrEP may be limited by perceptions of low risk from infrequent condomless sex with casual partners [27, 31], while it is likewise unreasonable to expect all PrEP-suitable people to want to take PrEP.

The final step of the PrEPARE cascade includes subjective benefits of PrEP use as a quality-of-life measure. Between 2017 and 2023, around three-quarters of PrEP users who had recently tested for HIV reported increased psychosexual wellbeing from PrEP, echoing benefits identified in the literature [35–37]. The proportion of the sample reporting increased psychosexual wellbeing from PrEP more than doubled over the same period, though the rate of increase slowed between 2021 and 2023, and it has not exceeded 80% relative to the previous cascade step. It may be that psychosexual wellbeing was already high among this group. However, it may also be that known barriers to continued PrEP use (e.g., cost and personal effort involved in maintaining use), difficulties with adherence, or other personal or relational issues suppress the potential benefits of PrEP for psychosexual wellbeing [38]. The scale items focused on reductions in HIV concern and increased sexual confidence from PrEP, and it is possible that PrEP does not uniformly achieve these outcomes for every user, and that by 2017, many PrEP users had already perceived a substantial decrease in HIV concern. These circumstances warrant further investigation.

There are limitations to our analyses. The GCPS and PrEPARE surveys used different sampling methods and operated in distinct contexts. The GCPS recruited participants primarily through community and event-based venues, and in most jurisdictions on a yearly basis, while PrEPARE relied solely on online recruitment every two years. Both surveys have unique strengths and weaknesses: as the GCPS have a shorter, field-adapted completion time, they attract much larger samples and are conducted more frequently, but they cannot explore topics in depth. The online, biennial format of PrEPARE means that topics can be explored in more depth, however the surveys attract fewer participants per round. We are unable to determine the representativeness of the convenience-based samples because there are limited national data on GBQ + people. PrEPARE participants may be more highly engaged or health literate and the study used PrEP-specific messaging in recruitment materials, which likely accounts for the greater level of PrEP use observed in that survey. PrEP suitability was operationalized differently in the two surveys due to differences in available variables. While this limits some direct comparisons between the two cascades, we believe their findings are complementary, identifying different potential points of intervention in similar samples. Both cascades applied consistent definitions over time, identifying patterns among GBQ + people that may be amenable to intervention to increase PrEP uptake. Lastly, it is possible that some individuals may have participated in the same survey over multiple rounds and/or across both studies. As the surveys were anonymous and did not include repeated measures or matched cases, it was not possible to assess how many people may have been repeat participants. However, as noted in the Methods, such repeat participation is most relevant to the interpretation of temporal trends and does not affect the interpretation of cross-sectional analyses conducted within the same time periods.

## Conclusion

Australia has made strong progress in PrEP implementation. Both PrEP cascades we developed showed that PrEP awareness has reached nearly ceiling levels and PrEP uptake has improved steadily over the past decade. Potential barriers persist in the intervening stages between PrEP awareness and uptake, such as access to government-subsidized healthcare, perceived need or risk of HIV, and the extent to which individuals experience psychosexual benefits from PrEP. Our findings are relevant to countries with mature PrEP programs where awareness is already high. In Australia, like similar countries, the challenge has shifted from raising awareness to ensuring easier, cheaper PrEP access, supporting provider engagement, and understanding barriers to sustained use. Our approach, which combines national behavioral surveillance data with more detailed psychosocial data, offers a practical model for tracking PrEP implementation across multiple dimensions of care, and comparing results from different sources. As new types of PrEP are introduced, programs will need flexible monitoring frameworks that can capture progress and guide timely policy responses.

## Supplementary Information

Below is the link to the electronic supplementary material.

Supplementary file1 (DOCX 43 KB)
