# Supplementary material for: A Decade of HIV Pre-Exposure Prophylaxis (PrEP) Cascade Monitoring: National Trends and Persistent Gaps in PrEP Use Among Gay, Bisexual and Queer Men and Non-Binary People in Australia
Source: AIDS Behav. 2026 Mar 17;30(10):3059–73. doi: 10.1007/s10461-026-05100-x (PMC13669015; doi:10.1007/s10461-026-05100-x)
Supplement: Supplementary file 1 [file 10461_2026_5100_MOESM1_ESM.docx]

**Supplemental Table 1.** Operationalization of PrEP suitability criteria

| Suitability criteria in 2023 Australian PrEP prescribing guidelines^1^ | Criteria adapted in GBQ+ Community Periodic Surveys | Criteria adapted in PrEPARE Project |
| --- | --- | --- |
| 1. CAI with a HIV-positive regular male partner (not on treatment and/or detectable viral load) in the last three months | CAI with a HIV-positive regular male partner (not on treatment and/or detectable viral load) in the last six months | CAI with a HIV-positive regular male partner (not on treatment and/or detectable or unknown viral load) in the last six months |
| 2. Any receptive CAI with any casual male partner in the last three months | Any receptive CAI with casual male partners in the last six months | Any CAI with casual male partners in the last six months |
| 3. Rectal gonorrhoea, rectal chlamydia or infectious syphilis diagnosis in the last three months | Any sexually transmissible infection diagnosis other than HIV in the last 12 months | Any sexually transmissible infection diagnosis other than HIV in the last 12 months |
| 4. Any sexualised drug use in the last three months (typically use of crystal methamphetamine or gamma-hydroxybutyrate) | Methamphetamine or gamma-hydroxybutyrate use in the last six months | Any drug use for the purpose of sex in the last six months |
| 5. Any anal intercourse where a condom broke or slipped off with a partner of unknown HIV status or they were HIV positive and not on treatment or had a detectable viral load |  |  |

^1^ The Australasian Society for HIV, Viral Hepatitis and Sexual Health Medicine (ASHM) National PrEP Guidelines Update. Prevent HIV by Prescribing PrEP. Sydney, 2023

Note. The 2023 guidelines were applied retrospectively across all years. The guidelines also indicate suitability if someone ‘foresees that there are likely to be similar [HIV] acquisition risks in the next 3 months’, however there were no corresponding survey measures assessing future risk. CAI = condomless anal intercourse.

**Supplemental Table 2.** Comparison of participants from the GCPS, 2022–23, and PrEPARE Project, 2023

|  | GCPS  N=9,177 | PrEPARE  N=1,907 | Unadjusted OR (95% CI) | *p* value | Adjusted OR (95% CI) | *p* value |
| --- | --- | --- | --- | --- | --- | --- |
| Age |  |  |  |  |  |  |
| 25 years and under | 1,289 (14.0) | 387 (20.3) | Ref |  | Ref |  |
| 26–35 | 3,318 (36.2) | 610 (32.0) | 0.61 (0.53–0.71) | <.001 | 0.50 (0.43-0.59) | <.001 |
| 36–55 | 3,311 (36.1) | 709 (37.2) | 0.71 (0.62–0.82) | <.001 | 0.60 (0.52-0.70) | <.001 |
| 56 years and over | 1,259 (13.7) | 201 (10.5) | 0.53 (0.44–0.64) | <.001 | 0.47 (0.38-0.57) | <.001 |
| State/territory |  |  |  |  |  |  |
| New South Wales | 3,276 (35.7) | 616 (32.3) | Ref |  | Ref |  |
| Queensland | 1,717 (18.7) | 353 (18.5) | 1.09 (0.95–1.26) | 0.222 | 1.01 (0.86-1.18) | .902 |
| South Australia | 812 (8.8) | 128 (6.7) | 0.84 (0.68–1.03) | 0.092 | 0.78 (0.63-0.97) | .025 |
| Victoria | 2,233 (24.3) | 531 (27.8) | 1.26 (1.11–1.44) | <.001 | 1.15 (1.01-1.32) | .035 |
| Western Australia | 866 (9.4) | 145 (7.6) | 0.89 (0.73–1.08) | 0.245 | 0.79 (0.64-0.97) | .026 |
| Other (ACT, NT & Tasmania) | 273 (3.0) | 134 (7.0) | 2.61 (2.09–3.27) | <.001 | 2.67 (2.10-3.40) | <.001 |
| Residential location |  |  |  |  |  |  |
| Inner metropolitan | 4,823 (52.6) | 919 (48.2) | Ref |  | Ref |  |
| Outer metropolitan | 2,578 (28.1) | 557 (29.2) | 1.13 (1.01–1.27) | 0.033 | 1.40 (1.23-1.59) | <.001 |
| Regional and remote | 1,404 (15.3) | 383 (20.1) | 1.43 (1.25–1.64) | <.001 | 1.69 (1.45-1.97) | <.001 |
| Postcode not provided | 372 (4.1) | 48 (2.5) | 0.68 (0.50–0.92) | 0.013 | 0.86 (0.62-1.18) | .348 |
| Born in Australia | 6,138 (66.9) | 1,337 (70.1) | 0.86 (0.77–0.96) | 0.006 | 0.90 (0.81-1.01) | .085 |
| Aboriginal and/or Torres Strait Islander | 256 (2.8) | 38 (2.0) | 0.71 (0.50–1.00) | 0.05 | 0.61 (0.43-0.87) | .007 |
| University degree | 5,576 (60.8) | 1,220 (64.0) | 1.14 (1.03–1.26) | 0.013 | 1.23 (1.09-1.37) | <.001 |
| Full-time employed | 6,420 (70.0) | 1,303 (68.3) | 0.93 (0.83–1.03) | 0.159 |  |  |
| Gay-identified | 7,205 (78.5) | 1,550 (81.3) | 0.86 (0.76–0.98) | 0.022 | 0.91 (0.80-1.05) | .198 |
| More than 10 male partners^1^ | 1,874 (20.4) | 452 (23.7) | 1.18 (1.05–1.32) | 0.006 | 0.83 (0.73-0.95) | .008 |
| Any condomless sex with regular partners^1^ | 4,927 (53.7) | 1,151 (60.4) | 1.31 (1.19–1.45) | <.001 | 1.00 (0.90-1.12) | .993 |
| Any condomless sex with casual partners^1^ | 3,886 (42.3) | 1,084 (56.8) | 1.79 (1.62–1.98) | <.001 | 1.39 (1.23-1.58) | <.001 |
| Recent HIV test^1^ | 5,070 (55.2) | 1,260 (66.1) | 1.58 (1.42–1.75) | <.001 | 0.77 (0.67-0.89) | <.001 |
| Any STI test^2^ | 5,840 (63.6) | 1,624 (85.2) | 3.28 (2.87–3.75) | <.001 | 3.45 (2.93-4.05) | <.001 |
| Any STI diagnosis^2^ | 1,945 (21.2) | 503 (26.4) | 0.93 (0.83–1.04) | 0.2 |  |  |
| Recent PrEP use^1^ | 3,750 (40.9) | 1,027 (53.9) | 1.59 (1.44–1.75) | <.001 | 1.10 (0.96-1.26) | .187 |

Data are n(%) except where stated. OR = odds ratio, CI = confidence interval, ACT = Australian Capital Territory, NT = Northern Territory, STI = sexually transmissible infection, PrEP = pre-exposure prophylaxis

**Supplemental Table 3.** Trends in PrEP suitability criteria among HIV-negative and untested GBQ+ people in the GCPS, 2014–2023

|  | 2014 | 2015 | 2016 | 2017 | 2018 | 2019 | 2020 | 2021 | 2022 | 2023 | Adjusted OR (95% CI) | *p* trend |
| --- | --- | --- | --- | --- | --- | --- | --- | --- | --- | --- | --- | --- |
|  | N=6,731 | N=7,328 | N=9,001 | N=8,592 | N=7,846 | N=8,508 | N=7,860 | N=6,413 | N=7,734 | N=8,365 | for year |  |
| CAI with a HIV-positive regular male partner not on treatment or with detectable viral load* | 9 (0.1) | 10 (0.1) | 11 (0.1) | 12 (0.1) | 4 (0.1) | 12 (0.1) | 11 (0.1) | 4 (0.1) | 5 (0.1) | 3 (0.0) | – | – |
| Receptive CAI with casual male partner* | 957 (14.2) | 1,040 (14.2) | 1,481 (16.5) | 1,940 (22.6) | 1,942 (24.8) | 2,372 (27.9) | 2,036 (25.9) | 1,702 (26.5) | 2,075 (26.8) | 2,747 (32.8) | 1.12 (1.12-1.14) | <.001 |
| Diagnosis with any STI other than HIV† | 702 (10.4) | 863 (11.8) | 1,208 (13.4) | 1,626 (18.9) | 1,600 (20.4) | 1,958 (23.0) | 1,621 (20.6) | 888 (13.8) | 1,208 (15.6) | 1,798 (21.5) | 1.08 (1.07-1.09) | <.001 |
| Any amphetamine or gamma-hydroxybutyrate use* | 787 (11.7) | 825 (11.3) | 978 (10.9) | 1,021 (11.9) | 837 (10.7) | 799 (9.4) | 819 (10.4) | 539 (8.4) | 583 (7.5) | 801 (9.6) | 0.96 (0.95-0.97) | <.001 |

Data are n(%) except where stated. Trends were not calculated for the first row due to small frequencies. CAI = condomless anal intercourse, OR = odds ratio, CI = confidence interval.

*In the last 6 months.

†In the last 12 months.

**Supplemental Table 4.** PrEP cascade for HIV-negative and untested GBQ+ people in the GCPS, 2014–2023

|  | 2014 | 2015 | 2016 | 2017 | 2018 | 2019 | 2020 | 2021 | 2022 | 2023 | Adjusted OR (95% CI) | *p* trend |
| --- | --- | --- | --- | --- | --- | --- | --- | --- | --- | --- | --- | --- |
|  | N=6,731 | N=7,328 | N=9,001 | N=8,592 | N=7,846 | N=8,508 | N=7,860 | N=6,413 | N=7,734 | N=8,365 | for year |  |
| PrEP suitable | 1,958 (29.1) | 2,150 (29.3) | 2,780 (30.9) | 3,183 (37.0) | 2,961 (37.7) | 3,462 (40.7) | 3,045 (38.7) | 2,209 (34.4) | 2,719 (35.2) | 3,647 (43.6) | 1.06 (1.06-1.07) | <.001 |
| Suitable and aware that PrEP is available | 574 (8.5) | 880 (12.0) | 1,472 (16.4) | 2,576 (30.0) | 2,592 (33.0) | 3,221 (37.9) | 2,817 (35.8) | 2,141 (33.4) | 2,612 (33.8) | 3,480 (41.6) | 1.20 (1.19-1.20) | <.001 |
| Suitable, aware, and using PrEP | 19 (0.3) | 47 (0.6) | 223 (2.5) | 924 (10.8) | 1,170 (14.9) | 1,808 (21.3) | 1,827 (23.2) | 1,248 (19.5) | 1,782 (23.0) | 2,487 (29.7) | 1.37 (1.36-1.38) | <.001 |
| Suitable, aware, using PrEP, and tested for HIV (last 6 months) | 16 (0.2) | 44 (0.6) | 211 (2.3) | 890 (10.4) | 1,141 (14.5) | 1,736 (20.4) | 1,724 (21.9) | 1,173 (18.3) | 1,658 (21.4) | 2,323 (27.8) | 1.36 (1.34-1.37) | <.001 |

Data are n(%). OR = odds ratio, CI = confidence interval.

**Supplemental Table 5.** Trends in PrEP suitability criteria among HIV-negative and untested GBQ+ people in the PrEPARE Project, 2017–2023

|  | 2017 | 2019 | 2021 | 2023 | Adjusted OR  (95% CI) | *p* trend |
| --- | --- | --- | --- | --- | --- | --- |
|  | N=1,038 | N=1,148 | N=1,185 | N=1,907 | for year |  |
| CAI with a HIV-positive regular male partner not on treatment or with detectable viral load* | 2 (0.2) | 3 (0.3) | 1 (0.1) | 1 (0.1) | – | – |
| Any CAI with casual male partner* | 473 (45.6) | 553 (48.2) | 628 (53.0) | 1,084 (56.8) | 1.10 (1.07-1.12) | <.001 |
| Diagnosis with any STI other than HIV† | 248 (23.9) | 326 (28.4) | 259 (21.9) | 503 (26.4) | 1.03 (1.01-1.06) | .029 |
| Any drug use for the purpose of sex in the last six months | 206 (19.8) | 204 (17.8) | 167 (14.1) | 290 (15.2) | 0.97 (0.93-0.99) | .015 |

Data are n(%) except where stated. Trends were not calculated for the first row due to small frequencies. CAI = condomless anal intercourse, OR = odds ratio, CI = confidence interval.

*In the last 6 months.

†In the last 12 months.

**Supplemental Table 6.** PrEP cascade for HIV-negative and untested GBQ+ people in the PrEPARE Project, 2017–2023

|  | 2017 | 2019 | 2021 | 2023 | Adjusted OR  (95% CI) | *p* trend |
| --- | --- | --- | --- | --- | --- | --- |
|  | N=1,038 | N=1,148 | N=1,185 | N=1,907 | for year |  |
| PrEP suitable | 568 (54.7) | 652 (56.8) | 687 (58.0) | 1,213 (63.6) | 1.08 (1.05-1.11) | <.001 |
| Aware | 552 (53.2) | 646 (56.3) | 677 (57.1) | 1,205 (63.2) | 1.09 (1.06-1.12) | <.001 |
| Willing to use PrEP | 390 (37.6) | 522 (45.5) | 565 (47.7) | 1,042 (54.6) | 1.14 (1.12-1.18) | <.001 |
| Discussed PrEP with a doctor | 291 (28.0) | 473 (41.2) | 518 (43.7) | 990 (51.9) | 1.20 (1.17-1.23) | <.001 |
| Ever used PrEP | 232 (22.4) | 435 (37.9) | 491 (41.4) | 962 (50.4) | 1.24 (1.21-1.28) | <.001 |
| Using PrEP* | 215 (20.7) | 384 (33.4) | 439 (37.0) | 876 (45.9) | 1.23 (1.20-1.27) | <.001 |
| Recently tested for HIV* | 209 (20.1) | 381 (33.2) | 428 (36.1) | 836 (43.8) | 1.22 (1.18-1.25) | <.001 |
| Increased psychosexual wellbeing from PrEP | 158 (15.2) | 267 (23.3) | 340 (28.7) | 648 (34.0) | 1.21 (1.17-1.25) | <.001 |

Data are n(%) except where stated. OR = odds ratio, CI = confidence interval.

*In the last 6 months.
